# When achalasia was not achalasia: occult esophagogastric junction squamous cell carcinoma revealed after failed peroral endoscopic myotomy

**DOI:** 10.1055/a-2984-7316

**Published:** 2026-11-04

**Authors:** Liuting Chen, Zhenxiang Wang, Huihui Sun, Ying Chen, Shuchang Xu

**Affiliations:** 1 Department of Gastroenterology 592018 Tongji Hospital Affiliated to Tongji UniversityShanghaiShanghaiChina

A 79-year-old woman presented with progressive postprandial vomiting for 6 months and a 4-kg weight loss within 2 weeks. Computed tomography revealed circumferential thickening of the middle and lower esophageal wall. Barium esophagography demonstrated a classic “bird-beak” narrowing at the distal esophagus. Initial esophagogastroduodenoscopy showed proximal esophageal dilation and resistance upon traversing the esophagogastric junction, with no other abnormalities noted in the esophagus or stomach ( Fig. 1 ). These findings favored a diagnosis of primary achalasia.

**Fig. 1:**
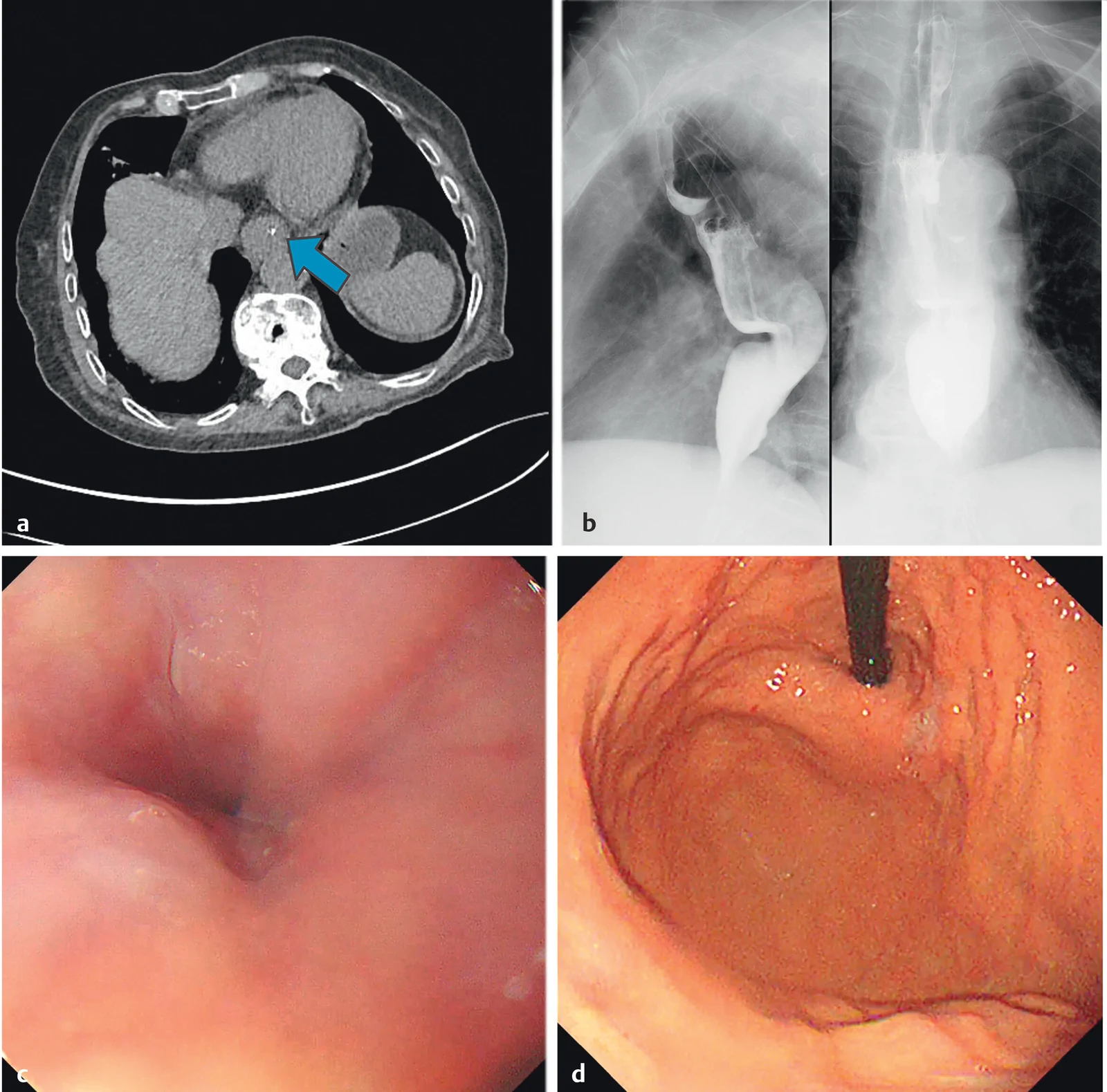
Initial endoscopic and imaging findings. ( **a** ) CT showing circumferential wall thickening of the mid-lower esophagus, with a high-density nasojejunal tube visible within the esophageal lumen (blue arrow). ( **b** ) A barium esophagography demonstrating classic “bird‑beak‑like” narrowing of the distal esophagus. ( **c** , esophagus) and ( **d** , stomach). EGD revealed proximal esophageal dilatation with resistance during passage across the esophagogastric junction; no additional lesions were identified within the esophagus or stomach.

Balloon dilation provided only transient symptom relief. The patient subsequently underwent peroral endoscopic myotomy (POEM). During the procedure, severe resistance was encountered at the stenotic segment, and submucosal tunneling revealed marked fibrosis with difficult tissue dissection ( Fig. 2 ).

**Fig. 2:**
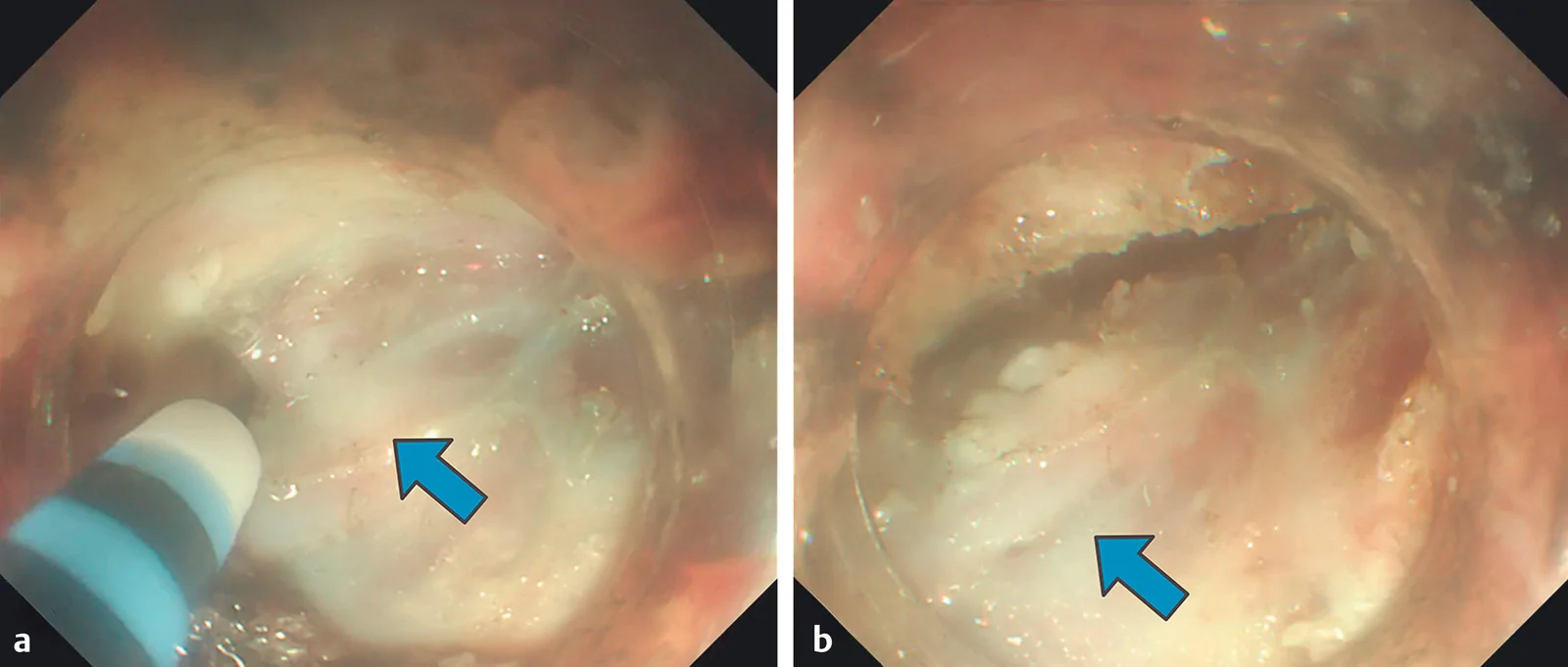
Endoscopy revealed fibrotic scar tissue in the submucosal tunnel, characterized by localized thickening and fibrosis of the submucosal layer (blue arrows).

After unsuccessful POEM, laparoscopic Heller myotomy and fundoplication were planned. Unexpectedly, laparoscopic exploration revealed an irregular, hard lesion at the distal esophagus and esophagogastric junction. Frozen-section examination confirmed malignancy, and radical resection was performed. Histopathological examination demonstrated moderately differentiated squamous cell carcinoma of the esophagogastric junction with multiple lymph node metastases ( Fig. 3 ).

**Fig. 3:**
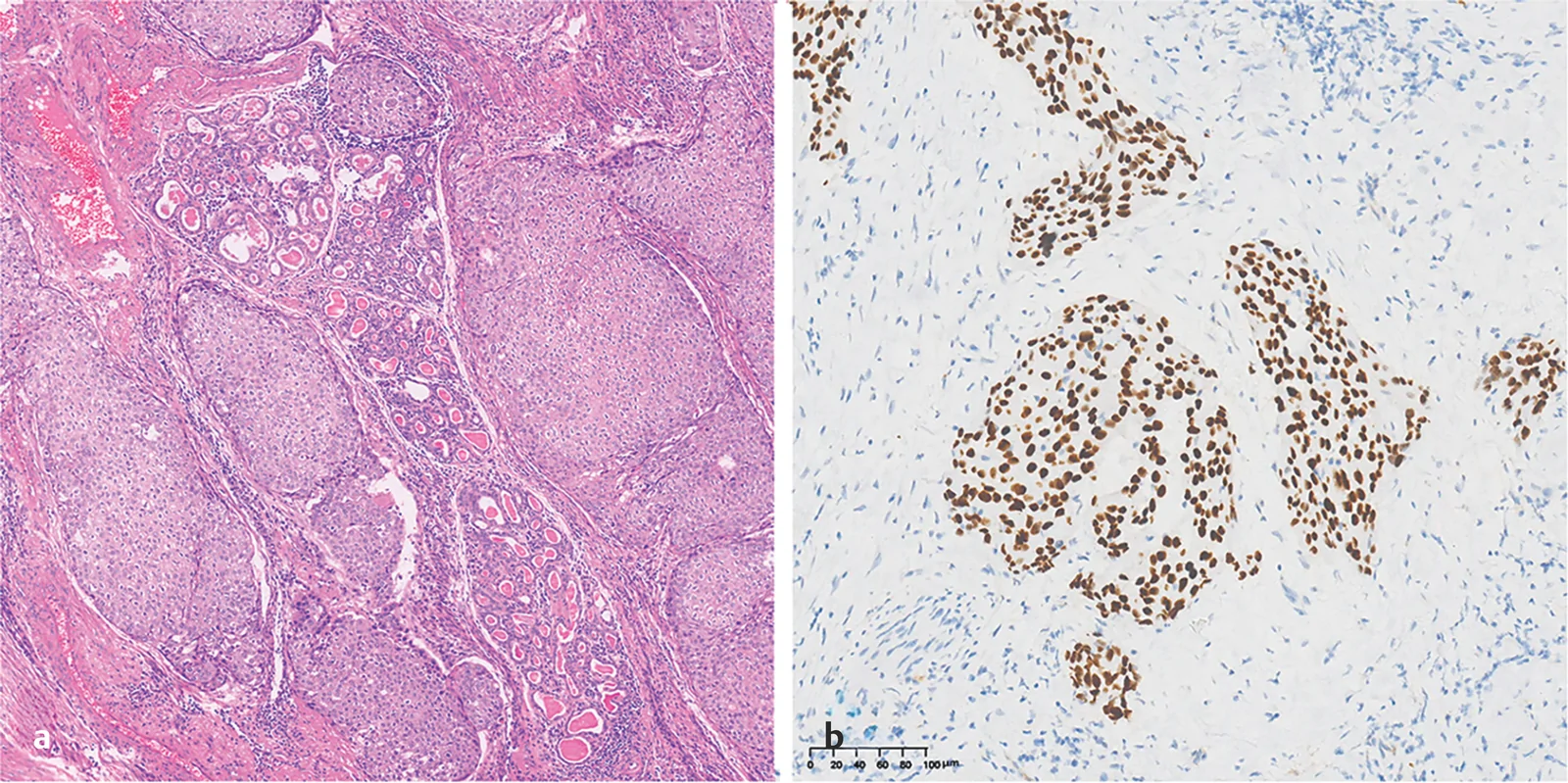
Pathology. ( **a** ) 20×, Hematoxylin and eosin stain. Squamous cell carcinoma of the esophagogastric junction. ( **b** ) 20×, p40 stain.

This case highlights that pseudoachalasia from occult malignancy can mimic primary achalasia despite extensive workup ( Video 1 ). Guidelines advise excluding secondary causes in patients aged >50 years with short duration, progressive dysphagia, and weight loss. 1​ However, infiltrating squamous cell carcinoma may lack a luminal mass, growing submucosally and muscularly, yielding normal mucosa and negative biopsies. 2​ Thus, normal endoscopy does not rule out malignancy, and persistent obstruction after myotomy or atypical POEM findings warrant re‑evaluation.

**Video 1** Key endoscopic and intraoperative findings of occult esophagogastric junction carcinoma mimicking achalasia. Here is the link to the video: .


Endoscopy_UCTN_Code_CCL_1AB_2AC_3AB

## Declaration of GenAI Use

Generative artificial intelligence was only used for minor language polishing. All contents are medically accurate and authors take full responsibility.
